# Supplementary material for: Landslide hazard early warning method for rock slopes using a hybrid LSTM-SARIMA data-driven model
Source: PLoS One. 2025 May 23;20(5):e0323650. doi: 10.1371/journal.pone.0323650 (PMC12101732; doi:10.1371/journal.pone.0323650)
Supplement: Attachment — Submitted filename: Response to Reviewers.docx [file pone.0323650.s016.docx]

**Responds to the reviewer’s comments:**

In the revised manuscript, we used the following color code/legend to distinguish changes in the revised manuscript:

- Changes made in reply to comments from Reviewer #1. (red)
- Changes made in reply to comments from Reviewer #2. (green)
- (If the different reviewers point out the same problem, use purple font to modify)

# To Reviewer 1:

**1. The title of the paper 'A Novel Method... Hybrid Data Driven Model' is too vague. Suggest modifying it to a specific method statement.**

- Response

We thank the experts for their valuable comments and we apologize for the unclear presentation of the title of the paper. In this paper, we mainly predict the slope displacement by LSTM-SARIMA hybrid data-driven model, which is combined with the early warning criterion of improving the tangential angle to complete the accurate early warning of slope landslide disaster.

The comments made by the experts are very valuable, therefore, we modify the title of the paper to “Landslide Hazard Early Warning System for Rock Slopes Using a Hybrid LSTM-SARIMA Data-Driven Model .”

- Revise

Thanks to the valuable advice of the experts, we have revised the title of the article.

| Title | |
| --- | --- |
| Original version | Revised version |
| A Novel Method for Landslide Risk Warning of Rock Slopes Based on a Hybrid Data-Driven Model | Landslide Hazard Early Warning Method for Rock Slopes Using a Hybrid LSTM-SARIMA Data-Driven Model |

2. **The authors may have confused the concept and difference of the specialized term “hazard” and “risk”. From the perspective of the main content, this work only involves “hazard”. Please refer to previous publications and follow the landslide risk assessment framework. For example: van Westen et al., 2006, https://doi.org/10.1007/s10064-005-0023-0, Wang et al., 2021, https://doi.org/10.3390/rs13132625，Wen et al., DOI: 10.1016/j.jenvman.2023.118177. Suggest modifying the relevant terms throughout the context, such as: the "risk" should be "hazard", the "Early warning" should be "Early hazard warning".**

- Response

Thanks for the Reviewers’ careful review. We are sorry for the paper's unclear presentation. In this paper, through the technical processes of data noise reduction, displacement component separation, slope displacement prediction, and early warning indicator establishment, we achieve accurate early warning of slope disasters. We are sorry that the expression of risk and hazard is not clear enough here, thanks to the valuable opinions of the experts, we have made corresponding modifications in many places in the paper to change the risk to hazard.

- Revise

In this article we will correct the terminology throughout the text by changing the word “risk” to “hazard”.The table below shows only the terminology changes in the first paragraph, but the changes have been made throughout the text.Changes are marked in red in the text.

| Full text | |
| --- | --- |
| Original version | Revised version |
| Mineral resources are important pillars for the survival and development of human society and guarantee national economic and social development. With the development of science and technology, mining is moving toward increasingly deeper levels, accompanied by the frequent occurrence of geological disasters (Song et al., 2023). Landslide disasters are among the most common geological disasters in the production process of mines, and the in-depth development of open-pit mine slope slippage early warning technology research to promote the efficient mining of mineral resources and reduce the loss of people and property in open-pit mining has important theoretical and practical significance (Yu et al., 2007, Li et al., 2023, Wang et al., 2014).  Landslide disaster early warning is a complex technical system that encompasses data preprocessing, including noise reduction and data cleaning, analysis of deformation influencing factors, selection of intelligent prediction algorithms, and the development of early warning guidelines. Each of these technologies represents a critical component within the landslide early warning system, with each element being interconnected and mutually supportive.The application of early warning technologies for landslide hazards enables the timely detection of precursory signs of landslide occurrence,and countermeasures can be taken in advance by evaluating the danger of slopes, which is currently considered to be the most effective technique for reducing the risk of landslides (Lin et al., 2009). | Mineral resources are important pillars for the survival and development of human society and guarantee national economic and social development. With the development of science and technology, mining is moving toward increasingly deeper levels, accompanied by the frequent occurrence of geological disasters (Wen et al., 2023). Landslide disasters are among the most common geological disasters in the production process of mines, and the in-depth development of open-pit mine slope slippage early hazard warning technology research to promote the efficient mining of mineral resources and reduce the loss of people and property in open-pit mining has important theoretical and practical significance (Westen et al., 2006, Li et al., 2021, Wang et al., 2014).  Landslide disaster early hazard warning is a complex technical system that encompasses data preprocessing, including noise reduction and data cleaning, analysis of deformation influencing factors, selection of intelligent prediction algorithms, and the development of early hazard warning guidelines. Each of these technologies represents a critical component within the landslide early hazard warning system, with each element being interconnected and mutually supportive.The application of early hazard warning technologies for landslide hazards enables the timely detection of precursory signs of landslide occurrence,and countermeasures can be taken in advance by evaluating the danger of slopes, which is currently considered to be the most effective technique for reducing the hazard of landslides (Lin et al., 2009). |

3. **The Introduction section of the manuscript does not clearly state the research gap, nor does it highlight the innovation and contribution of this work. Suggest adding the state-of-the-art literatures, such as: Doi: 10.1016/j.gsf.2024.101959, Doi: 10.1016/j.gr.2023.09.016, Doi: 10.1007/s11440-023-02050-9 to further clarify the research gap.**

- Response

Thanks for the Reviewers’ careful review. We are sorry for the paper's unclear presentation. The introductory part of this paper focuses on the research done by experts and scholars on slope displacement prediction in the past time, mainly focusing on both mathematical methods and artificial intelligence algorithms. For the research results of scholars in recent years are lacking, therefore, this paper adds the results taken by scholars in this direction in recent years.

- Revise

This paper will complement the research progress made by experts and scholars in recent years in the area of slope sliding.

| Line 111, Page 3 | |
| --- | --- |
| Original version | Revised version |
| There is no relevant description. | Recent studies have attempted to bridge this gap by integrating geomechanical insights with AI-based predictions. For instance, Xiang et al. (Xiang et al.2025) analyzed the failure mechanisms of step-like landslide displacements under rainfall and reservoir water level dynamics, providing a novel approach for integrating physical insights with predictive modeling . Similarly, Xiang et al. (Xiang et al.2024) proposed a hybrid support vector regression model optimized with factor preprocessing to enhance the prediction of step-like displacements in the Three Gorges Reservoir area, emphasizing the importance of unsaturated soil mechanics in deformation analysis. Furthermore, Wen et al. (Wen et al. 2023) developed a hybrid PSO-GSA-SVR model utilizing singular spectrum analysis to improve landslide displacement forecasting, demonstrating its effectiveness in the Jiuxianping landslide case study. |

4. **For displacement prediction models with periodic and trend terms, it should be further clarified how to divide the training and testing datasets.**

- Response

Thank you very much for this expert's valuable opinion, this opinion is of great value to improve the quality of our article. This paper lacks elaboration on dataset partitioning, therefore, we will explicitly state how the training and testing sets are partitioned in the paper.

- Revise

We will describe the method of dividing the training and test sets in the paper.

| Line 605, Page 24 | |
| --- | --- |
| Original version | Revised version |
| There is no relevant description. | After decomposing the displacement data into trend and periodic components, a total of 36,000 data groups were obtained for each type of displacement. Both datasets were split in an 8:2 ratio, with 80% allocated for training and 20% for testing. A hybrid data-driven model was then applied to analyze the displacement characteristics. |

5. **The author should further clarify the details of the study case, the rock slope and the monitoring data (displacement).**

- Response

Thanks for the Reviewers’ careful review. We are sorry for the paper's unclear presentation. Further details of the study cases, rocky slopes and monitoring data will be elucidated in the text.

- Revise

We will further elucidate the details of the study case, rocky slopes and monitoring data in this paper.

| Line 561, Page 21 | |
| --- | --- |
| Original version | Revised version |
| This paper presents 2 years of data monitoring and analysis work on a large open-pit mine in eastern China. The mine is located in the ancient uplift of the Mesozoic terrestrial volcanic basin margin. The main fracture systems in the mine area are three groups—northeast oriented, northwest oriented and nearly east–west oriented—all of which are manifested as the late stage of mineralization and play a destructive role in the ore body. Northeast-oriented fractures, which are characterized by compression and torsion, are the main fractures in mining areas and are characterized by long-term inheritance and development. The intersection of early northeast-oriented fractures and northwest-oriented fractures not only controls the formation of volcanic bodies but also provides tectonic space for the intrusion of subdiagonal granite porphyry rocks. The length of the strike is generally 500~2000 m, and the dip angle is generally 65~80°. There are F1, F2, F3, F4, F5, and F6 faults in the northeast direction. The present study area is mainly located in the local area of the F4 fault zone, which contains three levels of unincorporated sections of working steps with a height of 36 meters and a width of 25 meters. The distribution of major fracture structures in the mine area is shown in Figure 8. | This paper presents 2 years of data monitoring and analysis work on a large open-pit mine in eastern China. The mine is located in the ancient uplift of the Mesozoic terrestrial volcanic basin margin. The main fracture systems in the mine area are three groups—northeast oriented, northwest oriented and nearly east–west oriented—all of which are manifested as the late stage of mineralization and play a destructive role in the ore body. Northeast-oriented fractures, which are characterized by compression and torsion, are the main fractures in mining areas and are characterized by long-term inheritance and development. The intersection of early northeast-oriented fractures and northwest-oriented fractures not only controls the formation of volcanic bodies but also provides tectonic space for the intrusion of subdiagonal granite porphyry rocks. The length of the strike is generally 500~2000 m, and the dip angle is generally 65~80°. There are F1, F2, F3, F4, F5, and F6 faults in the northeast direction.  The rock slope under investigation is located on the southern side of the mining area and is nearly linear, with an average width of approximately 670m. According to the profile characteristics, the highest elevation is +121.50m, the lowest elevation is -350.00m, and the maximum height difference is 471.50m. The overall slope angle is approximately 41.32°. The lithology primarily consists of mixed gneiss and mixed granite. The slope is affected by a fault (F4) with a strike of SE60~70° and a dip angle of 75°. Layered joint structures are well-developed along the slope, with dip angles ranging from 30° to 60°. The rock mass structure is predominantly blocky.The field photographs of the rock slope and the corresponding profile are shown in Figure 8.  The mine utilizes an R/HYB2000 non-contact slope radar with an operational range of 5.5 km, a monitoring coverage of 110° × 42°, and a detection accuracy of no less than 0.1 mm. The radar is installed on the northern side of the open-pit mine, as shown in Figure 8, to monitor surface displacement changes on the southern slope. The monitoring data used in this study include slope displacement data obtained from radar and various sensors, as well as rainfall, groundwater level, and groundwater osmotic pressure data. Data were collected at a time interval of 30 minutes, resulting in a total of 36,000 monitoring datasets. |

6. **What are the limitations of this work comparing relevant previous publications? Need to do in-depth analysis in the discussion section.**

- Response

Many thanks to the experts for their valuable inputs. This comment is very helpful in improving the quality of the article. After describing the theory, methodology, and application, this article lacks discussion and comparison of research results. We will add a discussion section at the end of the article to clarify the effects and limitations of the research content.

- Revise

We will add a discussion section at the end of the article.

| Line 708, Page 30 | |
| --- | --- |
| Original version | Revised version |
| There is no relevant description. | **4.**Discussion  Although the proposed landslide early hazard warning framework has demonstrated significant improvements in prediction accuracy and practical applicability, several limitations remain when compared to previous studies. One of the main challenges is the dependency on high-quality monitoring data. While noise reduction techniques, such as sliding average and wavelet denoising, enhance data reliability, the effectiveness of the warning system can still be affected by missing or inconsistent data, particularly in extreme weather conditions or complex geological settings.  Furthermore, the hybrid AI-based model integrates physical insights with machine learning algorithms, improving prediction accuracy. However, the interpretability of AI-driven models remains a challenge. Unlike purely physics-based approaches, machine learning models often function as black boxes, making it difficult to directly correlate predicted outcomes with underlying geomechanical processes. Future research should focus on enhancing the explainability of AI models by incorporating physical constraints and causal relationships within the learning framework.  This study provides a systematic and practical landslide early hazard warning approach that combines monitoring, modeling, and intelligent prediction. Addressing the existing limitations will further enhance the robustness and applicability of this framework, contributing to more reliable and standardized early hazard warning systems in open-pit mining and other geotechnical settings. |

7. **There is no unified for the specialized terms. As shown in the legend in the upper right corner of Figure 10, "Cycle" may be "periodic". It should review the entire context and further polish the writing English.**

- Response

Thanks for the Reviewers’ careful review. We are sorry for the paper's unclear presentation. We will standardize terminology throughout the text.

- Revise

We will standardize the terminology throughout the text, and “cycle” should be expressed as “periodic”.

| Line 604, Page 24 | |
| --- | --- |
| Original version | Revised version |
|   Fig. 10 Results of separating the trend and periodic terms of displacement |   Fig. 10 Results of separating the trend and periodic terms of displacement |

# To Reviewer 2:

**The manuscript presents a hybrid approach to landslide early warning with significant potential for practical application. By implementing the following suggested revisions, the paper can be strengthened methodologically and provide more comprehensive insights into slope stability prediction.**

1. **While the paper compares four models, consider adding more machine learning models or ensemble techniques for a more comprehensive comparison.**

- Response

Thanks a lot to the experts for their valuable comments, this comment is extremely helpful in improving the quality of the article. In this paper, three machine learning models are used to do performance comparison with hybrid LSTM-SARIMA model. Based on the comments made by the experts, we added RNN and LightGBM, which are very widely used nowadays, for displacement prediction.

- Revise

In this paper, we have added two machine learning models for displacement prediction to the original.

| Line 637, Page 26 | |
| --- | --- |
| Original version | Revised version |
| With every half hour as a time step, the LSTM–SARIMA hybrid data-driven model was used to predict the trend term of displacement and the period term of displacement for the next 20 time steps, and the prediction results were summed to obtain the total slope displacement. To determine the excellence of the hybrid model, this paper uses the SVM, random forest and XGBoost models to predict the slope displacements for comparison. Figure 14 shows an error line plot of the displacement data obtained from the four data-driven model predictions plotted against the observed data on the day before the landslide. | With every half hour as a time step, the LSTM–SARIMA hybrid data-driven model was used to predict the trend term of displacement and the period term of displacement for the next 20 time steps, and the prediction results were summed to obtain the total slope displacement. To determine the excellence of the hybrid model, this paper uses the SVM, Random Forest, XGBoost, RNN and LightGBM models to predict the slope displacements for comparison. Figure 14 shows an error line plot of the displacement data obtained from the four data-driven model predictions plotted against the observed data on the day before the landslide.    (b) RNN    (e) LightGBM  Fig. 14 Error line diagrams for the 4 data-driven models |

2. **Elaborate on the limitations of the sliding average-wavelet noise reduction method and potential alternative approaches.**

- Response

Thanks for the Reviewers’ careful review. We are sorry for the paper's unclear presentation.We will detail the limitations and possible alternatives to the sliding average wavelet noise reduction method in this paper.

- Revise

We will add an elaboration related to the sliding average wavelet noise reduction method in the paper.

| Line 214, Page 6 | |
| --- | --- |
| Original version | Revised version |
| Denoising is applied to radar displacement monitoring data. Given the complexity and irregularity of this data, which is influenced by various factors, a single denoising method is insufficient to fully eliminate noise from the monitoring data. In this paper, the sliding average filter–wavelet denoising combined work method and noise reduction process are shown in Figure 2. Sliding average filtering for high-frequency noise has a better inhibitory effect, can smooth the signal, and can achieve comprehensive analysis of the global signal for overall denoising. The denoising wavelet transform can perform multiscale analysis in the time-frequency domain, making it possible to better capture the local characteristics of the signal. Therefore, the sliding average filtering-wavelet denoising method can be combined with global noise reduction and local focus noise reduction to effectively denoise monitoring data and provide excellent datasets for subsequent digital analysis. | Denoising is applied to radar displacement monitoring data. Given the complexity and irregularity of this data, which is influenced by various factors, a single denoising method is insufficient to fully eliminate noise from the monitoring data. In this paper, the sliding average filter–wavelet denoising combined work method and noise reduction process are shown in Figure 2. Sliding average filtering for high-frequency noise has a better inhibitory effect, can smooth the signal, and can achieve comprehensive analysis of the global signal for overall denoising. The denoising wavelet transform can perform multiscale analysis in the time-frequency domain, making it possible to better capture the local characteristics of the signal. Therefore, the sliding average filtering-wavelet denoising method can be combined with global noise reduction and local focus noise reduction to effectively denoise monitoring data and provide excellent datasets for subsequent digital analysis.It is worth noting that when combining moving average with wavelet denoising, there is an issue of parameter complexity. This complexity stems from the coupling of multiple parameters, the explosion of the search space, and conflicting objectives, which result in a reliance on empirical knowledge or the consumption of substantial resources for parameter tuning in practical applications. If computational resources are limited, alternative denoising algorithms, such as Kalman filtering or adaptive filtering, may be considered for data processing. |

3. **Provide a more in-depth theoretical explanation of how different environmental factors interact to influence slope displacement.**

- Response

Thanks for the Reviewers’ careful review. We are sorry for the paper's unclear presentation. The mechanism of the effect of rainfall, groundwater level, and groundwater infiltration pressure on slope displacement is complex but well documented. This paper is not clear enough about how different environmental factors interact to affect slope displacement, so we have rethought and reorganized our work in order to express the mechanism more clearly.

- Revise

We will provide a more in-depth theoretical explanation of how different environmental factors interact in order to affect slope displacements in the paper.

| Line 336, Page 12 | |
| --- | --- |
| Original version | Revised version |
| The cyclic term in mine slope displacements refers to displacements that vary cyclically due to the influence of external environmental factors such as rainfall, groundwater infiltration pressure, and groundwater level. These periodic changes often have different frequencies and amplitudes at different time scales, which have important effects on slope stability.  Rainfall is one of the most important factors leading to cyclic changes in the displacement of mine slopes. When the water content inside the soil slope increases dramatically during rainfall, the self-weight of the slope body increases, the shear strength of the soil body decreases significantly, and the pore water pressure increases, which are the main factors leading to the decrease in the stability of soil slopes. Rainfall water penetrates the rock body and changes the pore water pressure of the rock, which in turn affects the strength and stability of the rock. During the rainfall period, the permeability pressure on the rock mass increases, which may lead to an increase in slope displacement, while after the rainfall stops, the pore water in the rock mass is gradually emptied, the permeability pressure decreases, and the slope displacement may decrease or stabilize.  Cyclical changes in groundwater level are also among the important factors leading to cyclical changes in mine slope displacement. Seasonal changes, cyclical fluctuations or long-term trend changes in the groundwater level can affect the pore water pressure of the rock mass, which in turn affects the stability of the slope.  Moreover, the infiltration pressure of groundwater also has an impact on the stability of slopes. When the groundwater level rises, the infiltration pressure of groundwater increases, causing the hydraulic pressure on the slope to increase, thus exacerbating the slope displacement; in contrast, when the groundwater level falls, the infiltration pressure decreases, and the slope displacement may decrease or stabilize.  In addition to rainfall, the groundwater infiltration pressure and the water table, other factors, such as earthquakes, tectonic activity, and artificial mining, may also affect the cyclic displacement of mine slopes. These factors may lead to changes in the stress distribution within the rock mass and in the rock structure, which in turn may cause slope displacements. | The periodic term in mine slope displacements refers to periodic variations in displacement driven by external environmental factors such as rainfall, groundwater infiltration pressure, and groundwater level fluctuations. These factors interact in complex ways, influencing slope stability across different time scales through changes in stress distribution, pore pressure dynamics, and material strength.  Rainfall is a primary driver of periodic displacement in mine slopes, acting through multiple interconnected mechanisms. When rainfall infiltrates the soil or rock mass, it alters the internal moisture content, increasing the self-weight of the slope while simultaneously reducing the shear strength of the soil or weathered rock. The infiltration process also leads to a rise in pore water pressure, which decreases the effective stress within the slope and weakens its structural integrity. During intense or prolonged rainfall events, the rapid accumulation of pore water pressure can temporarily accelerate slope displacement. However, after rainfall ceases, drainage processes gradually reduce the excess pore pressure, leading to a temporary stabilization or even a reversal in displacement trends.  Groundwater level fluctuations further contribute to the periodic nature of slope displacements, often exhibiting seasonal or long-term trends. As the groundwater table rises, the increased hydrostatic pressure within rock fractures and pore spaces generates additional seepage forces, exerting outward pressure on the slope. This effect can induce progressive deformation, particularly in rock masses with pre-existing discontinuities or weak structural planes. Conversely, when the groundwater level declines, the reduction in hydraulic pressure may relieve some of the stress within the slope, leading to a decrease or stabilization in displacement rates. However, in cases where desaturation causes a loss of matric suction in unsaturated soil layers, the slope may experience delayed or residual deformation, further complicating displacement patterns.  Groundwater infiltration pressure plays a critical role in slope displacement, particularly in fractured rock masses where water movement is highly anisotropic. Increased infiltration pressure due to rising groundwater levels or prolonged rainfall events can exacerbate shear stress along weak planes, promoting progressive failure. Additionally, variations in permeability between different geological layers may lead to differential pressure buildup, further influencing deformation patterns. In contrast, a drop in groundwater infiltration pressure due to seasonal drying or dewatering activities can reduce the driving forces acting on the slope, potentially enhancing its stability.  Beyond hydrological influences, external factors such as seismic activity, tectonic stress changes, and mining operations also modulate periodic displacement behavior. Seismic events generate transient dynamic loads, which can induce immediate displacement shifts or trigger long-term weakening effects through stress redistribution. Similarly, tectonic activity can introduce gradual deformation trends, interacting with hydrological processes to create complex displacement periods. Mining-induced stress redistribution, particularly in open-pit operations, alters the local stress field and can lead to incremental slope movement, further compounding the effects of environmental fluctuations.  The interaction of these factors underscores the complexity of periodic slope displacement mechanisms. Rather than acting in isolation, rainfall, groundwater dynamics, and external geophysical forces collectively shape displacement trends through interdependent processes. A deeper understanding of these interactions is essential for refining predictive models and improving the reliability of landslide early hazard warning systems. |

4. **Include a section discussing the interpretability of the LSTM-SARIMA hybrid model, explaining how it captures complex nonlinear relationships.**

- Response

Thanks for the Reviewers’ careful review. We are sorry for the paper's unclear presentation. Therefore, we have added a subsection to the text dedicated to the section on the interpretability of the LSTM-SARIMA hybrid model, explaining how it captures complex nonlinear relationships.

- Revise

We will add a subsection to the text that discusses the interpretability of the LSTM-SARIMA hybrid model in the section.

| Line 428, Page 16 | |
| --- | --- |
| Original version | Revised version |
| There is no relevant description. | 2.2.4 LSTM-SARIMA hybrid model  The LSTM-SARIMA hybrid model demonstrates significant advantages by combining the powerful nonlinear modeling capability of LSTM with the excellent handling of seasonality and trends provided by SARIMA. LSTM excels in capturing long-term dependencies and nonlinear relationships within time series data, dynamically adjusting the state of its memory units to capture complex patterns and abrupt changes in slope displacement. This enables it to account for nonlinear impacts caused by sudden environmental changes or unexpected events, such as blasting vibrations, earthquakes, or shifts in mining activities, which can lead to sharp variations in displacement patterns that traditional linear models often fail to capture. However, through its adaptive learning ability, LSTM can automatically learn these complex patterns from historical data, enhancing the model's accuracy and flexibility. Although LSTM is inherently a black-box model, visualizing its internal states (such as weights and activation values) and performing local sensitivity analysis can help identify which input features (such as lithology, weathering degree, etc.) play a significant role in predicting displacement.  On the other hand, SARIMA effectively captures linear trends and seasonal characteristics within time series, providing a stable baseline prediction for the model. SARIMA is capable of handling long-term periodic changes, such as precipitation periodic and temperature fluctuations, and can also identify linear trends, such as the gradual changes in slope stability over time. This feature allows SARIMA to offer a robust foundation for the model by explicitly accounting for seasonal and trend components that explain long-term variations in the time series. Since SARIMA relies on linear relationships, its coefficients are directly interpretable. The seasonal and trend components of the model can help explain periodic variations and long-term stability trends in slope displacement.  By combining the nonlinear modeling capabilities of LSTM with the linear modeling strengths of SARIMA, the hybrid model can simultaneously handle both short-term sudden events and long-term seasonal changes when facing complex slope displacement variations. This results in a more comprehensive description of the dynamic changes in slope displacement. This integration makes the LSTM-SARIMA hybrid model not only highly accurate in predicting slope displacement but also provides more intuitive explanatory support for engineering practice. |

5. **Enhance the manuscript with more detailed visualizations, particularly showing the intermediate steps of data processing and model prediction.**

- Response

Thanks for the Reviewers’ careful review. We are sorry for the paper's unclear presentation. We fully understand the importance of presenting the intermediate steps of data processing and model prediction in detail, however, the inherent nature of machine learning models (especially deep learning architectures), whose internal feature representations are often automatically generated through multiple layers of nonlinear transformations, makes it notably challenging to visualize the intermediate processes. Unlike white-box models, the feature extraction paths of such models are difficult to decouple into interpretable discrete steps, and their prediction mechanisms rely more on distributed representations than on manually predefined logical rules.

In order to enhance the transparency of the methodology used in this paper, we provide relatively detailed descriptions of the mechanism, characteristics and effects of the models in Articles 2.2.1, 2.2.2 and 2.2.3, and make use of Fig. 3, Fig. 4 and Fig. 5 to provide an obvious demonstration of the computational process of various models.

In practical application, we utilize Fig. 9 to demonstrate the comparison of model effects before and after noise reduction. We also demonstrate the results of model prediction using Fig. 10 to Fig. 11, and plot the error line graph (Fig. 14) to show the performance of different machine learning models.

Once again, we thank the experts for their valuable comments. We are aware that the current visualization presentation is still lacking. However, the characteristics of the model prevented us from proceeding further. We kindly request the experts for their understanding.

6. **Develop a more comprehensive section explicitly discussing the study's limitations and potential sources of error.**

- Response

Thanks for the Reviewers’ careful review. We are sorry for the paper's unclear presentation. We will explicitly discuss the limitations of the study and potential sources of error based on a comparison of existing research results and the findings of this paper.

- Revise

We will add a discussion section to the text that explicitly discusses the limitations of the study and potential sources of error.

| Line 708, Page 30 | |
| --- | --- |
| Original version | Revised version |
| There is no relevant description. | **4.**Discussion  Although the proposed landslide early hazard warning framework has demonstrated significant improvements in prediction accuracy and practical applicability, several limitations remain when compared to previous studies. One of the main challenges is the dependency on high-quality monitoring data. While noise reduction techniques, such as sliding average and wavelet denoising, enhance data reliability, the effectiveness of the warning system can still be affected by missing or inconsistent data, particularly in extreme weather conditions or complex geological settings.  Furthermore, the hybrid AI-based model integrates physical insights with machine learning algorithms, improving prediction accuracy. However, the interpretability of AI-driven models remains a challenge. Unlike purely physics-based approaches, machine learning models often function as black boxes, making it difficult to directly correlate predicted outcomes with underlying geomechanical processes. Future research should focus on enhancing the explainability of AI models by incorporating physical constraints and causal relationships within the learning framework.  This study provides a systematic and practical landslide early hazard warning approach that combines monitoring, modeling, and intelligent prediction. Addressing the existing limitations will further enhance the robustness and applicability of this framework, contributing to more reliable and standardized early hazard warning systems in open-pit mining and other geotechnical settings. |

7. **Furthermore, the work would greatly benefit from including and referencing more recent material on practical implementations of machine learning methods across several domains. It is recommended to engage in discussions on the comparison of results and the integration of these concepts into your work. You may find more information on this topic in the following articles: https://doi.org/10.1007/s11004-023-10116-3, https://doi.org/10.1007/s11069-024-06917-2, https://doi.org/10.1007/s12583-021-1525-9, https://doi.org/10.1007/s12583-021-1407-1.**

- Response

Thanks for the Reviewers’ careful review. We are sorry for the paper's unclear presentation. The introductory part of this paper focuses on the research done by experts and scholars on slope displacement prediction in the past time, mainly focusing on both mathematical methods and artificial intelligence algorithms. For the research results of scholars in recent years are lacking, therefore, this paper adds the results taken by scholars in this direction in recent years.

- Revise

This paper will complement the research progress made by experts and scholars in recent years in the area of slope sliding.

| Line 122, Page 3 | |
| --- | --- |
| Original version | Revised version |
| There is no relevant description. | Recent studies have attempted to bridge this gap by integrating geomechanical insights with AI-based predictions. For instance, Xiang et al. (Xiang et al.2025) analyzed the failure mechanisms of step-like landslide displacements under rainfall and reservoir water level dynamics, providing a novel approach for integrating physical insights with predictive modeling . Similarly, Xiang et al. (Xiang et al.2024) proposed a hybrid support vector regression model optimized with factor preprocessing to enhance the prediction of step-like displacements in the Three Gorges Reservoir area, emphasizing the importance of unsaturated soil mechanics in deformation analysis. Furthermore, Wen et al. (Wen et al. 2023) developed a hybrid PSO-GSA-SVR model utilizing singular spectrum analysis to improve landslide displacement forecasting, demonstrating its effectiveness in the Jiuxianping landslide case study. Lei et al. ( Lei et al.2024) proposed a method based on Bayesian ensemble learning and Shapley additive explanations for rapid slope stability estimation, and developed a user-friendly graphical interface tool to simplify the assessment process. Long et al. (Long et al. 2023) compared the application of supervised classification methods in landslide evolution research in the Mianyu River basin, China, and found that the Random Forest algorithm performed excellently in landslide identification. Additionally, Ma et al. (Ma et al. 2024) introduced an automated machine learning (AutoML) technique to propose an easy-to-use landslide susceptibility mapping method that enhances model accuracy and user-friendliness through automation. Other studies have shown that selecting appropriate unit types significantly affects the results of landslide susceptibility mapping. Ma et al. (2023) compared the application of slope units and grid units in landslide events in Gansu Province, China, and found that slope units effectively reduced model uncertainty, thereby improving prediction accuracy.These studies provide new methodologies and perspectives for landslide displacement prediction, particularly in addressing the nonlinear and complex behavior of landslides. By combining artificial intelligence algorithms with factor preprocessing techniques, these approaches have improved prediction accuracy and reliability. However, current research still faces challenges, including the lack of a unified early hazard warning standard and insufficient integration of monitoring data with deformation mechanisms. Therefore, further developments require establishing a closer connection between physical mechanism analysis and data-driven models to enhance the accuracy and practicality of landslide early hazard warning systems. |

8. **“Malery et al. (Malamud et al., 2010) Li Tianbin et al. (Chen et al., 2016)”: List only the first author's name followed by “et al.” in every citation.**

- Response

Thanks for the Reviewers’ careful review. We are sorry for the paper's unclear presentation.We have changed the corresponding expression in the text.

- Revise

We will revise the formulation of the citation in the text.

| Line 71, Page 1 | |
| --- | --- |
| Original version | Revised version |
| Malery et al. (Malamud et al., 2010) used mathematical statistics and probabilistic models to study the spatial and temporal distributions of landslides and developed many mathematical models to describe landslide phenomena. Li Tianbin et al. (Chen et al., 2016) established the Fernhas inverse function model based on the cumulative displacement‒time curve of landslides being opposite of the Fernhas curve. | Malamud et al. (Malamud et al., 2010) used mathematical statistics and probabilistic models to study the spatial and temporal distributions of landslides and developed many mathematical models to describe landslide phenomena. Li Tianbin et al. (Li Tianbin et al., 2016) established the Fernhas inverse function model based on the cumulative displacement‒time curve of landslides being opposite of the Fernhas curve. |

9. **More details of the open-pit mine slope should be provided. Picture and cross-section should be included.**

- Response

Thanks for the Reviewers’ careful review. We are sorry for the paper's unclear presentation. Further details of the study cases, rocky slopes and monitoring data will be elucidated in the text.

- Revise

We will further elucidate the details of the study case, rocky slopes and monitoring data in this paper.

| Line 548, Page 21 | |
| --- | --- |
| Original version | Revised version |
| This paper presents 2 years of data monitoring and analysis work on a large open-pit mine in eastern China. The mine is located in the ancient uplift of the Mesozoic terrestrial volcanic basin margin. The main fracture systems in the mine area are three groups—northeast oriented, northwest oriented and nearly east–west oriented—all of which are manifested as the late stage of mineralization and play a destructive role in the ore body. Northeast-oriented fractures, which are characterized by compression and torsion, are the main fractures in mining areas and are characterized by long-term inheritance and development. The intersection of early northeast-oriented fractures and northwest-oriented fractures not only controls the formation of volcanic bodies but also provides tectonic space for the intrusion of subdiagonal granite porphyry rocks. The length of the strike is generally 500~2000 m, and the dip angle is generally 65~80°. There are F1, F2, F3, F4, F5, and F6 faults in the northeast direction. The present study area is mainly located in the local area of the F4 fault zone, which contains three levels of unincorporated sections of working steps with a height of 36 meters and a width of 25 meters. The distribution of major fracture structures in the mine area is shown in Figure 8. | This paper presents 2 years of data monitoring and analysis work on a large open-pit mine in eastern China. The mine is located in the ancient uplift of the Mesozoic terrestrial volcanic basin margin. The main fracture systems in the mine area are three groups—northeast oriented, northwest oriented and nearly east–west oriented—all of which are manifested as the late stage of mineralization and play a destructive role in the ore body. Northeast-oriented fractures, which are characterized by compression and torsion, are the main fractures in mining areas and are characterized by long-term inheritance and development. The intersection of early northeast-oriented fractures and northwest-oriented fractures not only controls the formation of volcanic bodies but also provides tectonic space for the intrusion of subdiagonal granite porphyry rocks. The length of the strike is generally 500~2000 m, and the dip angle is generally 65~80°. There are F1, F2, F3, F4, F5, and F6 faults in the northeast direction.  The rock slope under investigation is located on the southern side of the mining area and is nearly linear, with an average width of approximately 670m. According to the profile characteristics, the highest elevation is +121.50m, the lowest elevation is -350.00m, and the maximum height difference is 471.50m. The overall slope angle is approximately 41.32°. The lithology primarily consists of mixed gneiss and mixed granite. The slope is affected by a fault (F4) with a strike of SE60~70° and a dip angle of 75°. Layered joint structures are well-developed along the slope, with dip angles ranging from 30° to 60°. The rock mass structure is predominantly blocky.The field photographs of the rock slope and the corresponding profile are shown in Figure 8.  The mine utilizes an R/HYB2000 non-contact slope radar with an operational range of 5.5 km, a monitoring coverage of 110° × 42°, and a detection accuracy of no less than 0.1 mm. The radar is installed on the northern side of the open-pit mine, as shown in Figure 8, to monitor surface displacement changes on the southern slope. The monitoring data used in this study include slope displacement data obtained from radar and various sensors, as well as rainfall, groundwater level, and groundwater osmotic pressure data. Data were collected at a time interval of 30 minutes, resulting in a total of 36,000 monitoring datasets.    （b）Rock slope site photos    （c）Engineering geological profile  Fig. 8 Study the details of the area |

10. **Provide more details about the data collection process, including the specific radar monitoring equipment used, installed location, its precision, and calibration methods.**

- Response

Thanks for the Reviewers’ careful review. We are sorry for the paper's unclear presentation.We will add to the text an elaboration on data acquisition, including the specific radar monitoring equipment used, installation locations, accuracy, and calibration methods.

- Revise

We will add an elaboration on data collection in the text.

| Line 570, Page 21 | |
| --- | --- |
| Original version | Revised version |
| There is no relevant description. | The mine utilizes an R/HYB2000 non-contact slope radar with an operational range of 5.5 km, a monitoring coverage of 110° × 42°, and a detection accuracy of no less than 0.1 mm. The radar is installed on the northern side of the open-pit mine, as shown in Figure 8, to monitor surface displacement changes on the southern slope. The monitoring data used in this study include slope displacement data obtained from radar and various sensors, as well as rainfall, groundwater level, and groundwater osmotic pressure data. Data were collected at a time interval of 30 minutes, resulting in a total of 36,000 monitoring datasets.    （a）Schematic diagram of the distribution of major fracture structures in the mine area and the location of the radar arrangement  Fig. 8 Study the details of the area |

11. **Fig. 16: please include the landslide boundary.**

- Response

Thanks for the Reviewers’ careful review. We are sorry for the paper's unclear presentation.We will show the landslide boundary.

- Revise

We will show the landslide boundary.

| Line 697, Page 29 | |
| --- | --- |
| Original version | Revised version |
|   Fig.16 Landslide site at an open pit mine in eastern China |   Fig.16 Landslide site at an open pit mine in eastern China |

12. **Conduct a thorough proofreading of the manuscript to correct any grammatical errors, improve sentence structure, and enhance overall clarity.**

- Response

Thanks for the Reviewers’ careful review. We apologize for the grammatical errors in the article. We will be checking and revising the entire article to correct all grammatical errors, improve sentence structure, and improve overall clarity.

- Revise

We will check and make corrections to grammar and structure throughout the text.

Full text
